# Cytokinin-Induced Parthenocarpic Fruit Development in Tomato Is Partly Dependent on Enhanced Gibberellin and Auxin Biosynthesis

**DOI:** 10.1371/journal.pone.0070080

**Published:** 2013-07-29

**Authors:** Jiangang Ding, Biwei Chen, Xiaojian Xia, Weihua Mao, Kai Shi, Yanhong Zhou, Jingquan Yu

**Affiliations:** 1 Department of Horticulture, Zhejiang University, Hangzhou, People’s Republic of China; 2 Key Laboratory of Horticultural Plants Growth, Development and Quality Improvement, Ministry of Agricultural, Hangzhou, People’s Republic of China; 3 School of Life Science and Engineering, Southwest University of Science and Technology, Mianyang, People’s Republic of China; Wake Forest University, United States of America

## Abstract

Fruit set of plants largely depends on the biosynthesis and crosstalk of phytohormones. To date the role of cytokinins (CKs) in the fruit development is less understood. Here, we showed that parthenocarpic fruit could be induced by 1-(2-chloro-4-pyridyl)-3-phenylurea (CPPU, an active CK) in tomato ( *Solanum* *lycopersicum* cv. Micro-Tom). The fresh weight of CPPU-induced parthenocarpic fruits was comparable with that induced by GA_3_. Importantly, CPPU-induced parthenocarpy was found to be compromised by simultaneous application of paclobutrazol (a GA biosynthesis inhibitor), and this effect could be restored by exogenous GA_3_. Like pollination, CPPU-induced fruit showed enhanced accumulation of GA_1+3_ and indole-3-acetic (IAA), which were accompanied by elevated expression of GA biosynthesis genes like *SlGPS, SlGA20ox1, SlGA20ox2* and *SlGA3ox1*, and IAA biosynthesis gene *ToFZY*. Elevated GAs level in CPPU-induced fruits was also associated with down-regulation of GA inactivation genes, namely *SlGA2ox1,2,3,4,5* in comparison with untreated control. These results suggested that CKs may induce parthenocarpy in tomato partially through modulation of GA and IAA metabolisms.

## Introduction

Fruit set is a vital phase of plant development encompassing transition of quiescent ovaries to fertilized and fully grown fruits [1–3]. It is usually achieved through successful pollination and fertilization or via parthenocarpy [1]. Parthenocarpy is an important physiological event and largely depends on the coordinated action of inherent hormones produced in the unpollinated ovaries [4,5]. Enhanced biosynthesis of auxins (Aux), gibberellins (GAs) and cytokinins (CKs) has been observed in pollinated ovaries and in the ovaries of the parthenocarpic mutants such as *pat*, *pat2* and *pat3/pat4*, in tomato [3,6–9]. Transcripts of the Aux and GAs responsive genes were up-regulated within 48 h after pollination in tomato ovaries, thereby suggesting the induction of Aux and GA signaling pathways by pollination [6]. Several lines of evidences showed that Aux and GAs are critically involved in fruit set and fruit development in tomato plants [7,10–12]. In practice, Aux and GAs are widely used to improve the fruit set under suboptimal growth conditions, which limits pollination and/or fertilization in commercial production of horticultural crops [1,8,13].

Pollination is usually associated with an increased level of GAs, which is accompanied by an induction of GA biosynthesis genes CPS (copalyldiphosphate synthase) and GA20-oxidase genes in tomato ovaries [9,14]. Fruit set in both pollinated wild-type and unpollinated parthenocarpic mutant tomato ovaries was reduced by the application of GA biosynthesis inhibitors, and this effect was found to be reversed by the exogenous application of GA_3_ [7,14]. On the other hand, overexpression of Aux biosynthesis genes or loss of function of components in the Aux signaling cascade showed altered parthenocarpic capacity [15,16]. A recent study elucidated that Aux could induce fruit set and growth in tomato, at least in part through enhancing GA biosynthesis, probably by decreasing GA inactivation activity [17]. It appears that the crosstalk between Aux and GAs plays a critical role in the fruit development.

Besides GAs and Aux, other phytohormones such as CKs, abscisic acid (ABA) and brassinosteroids (BRs) have also been shown to play roles in fruit set and subsequent fruit growth [18–20]. For instance, CKs play a critical role in the stimulation of cell division throughout the plant life cycle. In agreement with the concomitant increase in CKs level, transcripts of the CK biosynthesis genes was induced by pollination in tomato ovaries [3,6,21–23]. Interestingly, the effects of different hormones on the induction of fruit set vary with plant species. For example, Aux and GAs had less potential to induce parthenocarpic fruit growth than CKs in crops such as cucumber, kiwi fruit, melon and pear [24–29]. Work in our laboratory revealed that parthenocarpy induced by CKs and BRs was associated with increased transcripts of *CyCD3*, a gene involved in the cell cycle in plants [18,30]. However, lack of genomic information and mutant resource largely hinders the elucidation of the mechanism of parthenocarpy in these plant species. Additionally, the molecular mechanism by which CKs crosstalk with other hormones in the early phase of fruit development also remains elusive.

Although increased CKs accumulation was observed at the early stage of fruit development in tomato [3], the potential role of CKs in fruit set has not been clarified. Keeping this in mind, the present study hypothesizes that CKs may induce parthenocarpic growth via altering the endogenous metabolic profiles of GAs and Aux in tomato ovaries. To validate the above hypothesis, dynamics of endogenous GAs and indole-3-acetic acid (IAA) and the expression patterns of key genes of GAs and IAA metabolism in tomato ovaries after pollination or CKs treatment were examined. Our results showed that CKs-induced parthenocarpic fruit growth could be partly attributed to the altered metabolism of GA and IAA.

## Materials and Methods

### Plant material and growth conditions

*Solanum* *lycopersicum* cv. Micro-Tom plants were used in the present study. Seeds were obtained from the Tomato Genetics Resource Center at University of California (Davis). The seeds were germinated in trays filled with peat and vermiculite (1:1, v/v). On the appearance of the first two truly expanded leaves, seedlings were transplanted into plastic pots (one seedling/ pot, 15 cm diameter and 15 cm deep) containing the same medium. Seedlings were kept in growth chambers and watered with half-strength Hoagland’s solution. The growth conditions were as follows: temperature 25/17°C (day/night), a 12-h photoperiod, and light intensity of 200 µmol m^–2^ s^-1^. Only one flower per truss and the first two were left per plant for the experiment, as previously described [17]. All the unselected flowers were removed 2 days before anthesis.

### Hormone treatments and sampling

Flowers were emasculated 2 days before anthesis to prevent self-pollination except those used for the pollination treatment. *t*-Zeatin (ZT, Sigma-Aldrich, USA), 1-(2-chloro-4-pyridyl)-3-phenylurea (CPPU, Sigma-Aldrich, USA) and GA_3_ (Sigma-Aldrich, USA) dissolved in a solution containing 5% ethanol and 0.1% Tween 80 were applied to unpollinated (UP) ovaries at the defined concentrations, 10 µl per ovary on the day equivalent to anthesis. Paclobutrazol (PAC, Sigma-Aldrich, USA) was applied to the roots in the nutrient solution at 10^-5^ M at the rate of 200 ml per plant every 2 days, as previously described [12,17]. Ovaries treated with the solvent alone were set as unpollinated control. At 1, 3, 5, 10 and 20 days after anthesis (DAA), ovaries were collected, frozen in liquid nitrogen, and stored at -80°C respectively, until further analysis.

### Light microscopy and morphometric analyses

Fruits were sampled at 20 DAA. The pericarp was fixed in a mixture of 70% ethanol, formaldehyde, and acetic acid (90:5:5 by volume). A 5-mm-thick slice was cut out from the pericarp and embedded in paraffin. The transverse sections of 9-µm-thick were prepared from slices using microtome. The sections were mounted, stained with toluidine blue, and photographed under an Olympus motorized system microscopes (BX61, Olympus Co., Tokyo, Japan).

### Analysis of gibberellin (GA_1+3_) and IAA contents by ELISA

The extraction, purification and immunoassay of GA_1+3_ and IAA were performed using GA_1+3_ and IAA ELISA kit (China Agricultural University, Beijing, China) as described previously [29,31,32]. In brief, approximately 0.2 g fresh weight (FW) of ovaries or 2 g of fruits at different developmental stages was used for GAs and IAA extraction.

### Gene expression analysis

Transcript levels of genes involved in GA biosynthesis (*SlCPS*, *SlGPS*, *SlGA20ox1*, *SlGA20ox2*, *SlGA20ox3*, *SlGA20ox4*, *SlGA3ox1* and *SlGA3ox2*), GA catabolism (*SlGA2ox1*, *SlGA2ox2*, *SlGA2ox3*, *SlGA2ox4* and *SlGA2ox5*) and signaling (*SlGA INSENSITIVE DWARF1* (*SlGID1*)), and GA-inducible genes such as *SlGibberellin-stimulated transcripts1* (*SlGAST1*) and *SlDELLA* were analyzed with quantitative Real Time-Polymerase Chain Reaction (qRT-PCR). The primers used for corresponding genes were designed as described by Serrani et al [17]. The primer of IAA biosynthesis gene *ToFZY* (a tomato gene encoding a flavin monooxygenase involved in tryptophan-dependent Aux biosynthesis pathway) was designed according to Mariotti et al [3]. All these primers used were listed in Table S1. The expression of all the genes was analyzed by qRT-PCR analysis.

Total RNA was extracted from the ovaries/fruits using the Trizol reagent according to the supplier’s instructions. Total RNA was reverse-transcribed using ReverTra Ace qPCR RT Kit (Toyobo, Japan) following the supplier’s recommendation. qRT-PCR was performed using the iCycler iQ™ Real-time PCR Detection System (Bio-Rad, Hercules, USA) using iQ SYBR Green SuperMix (Takara, Japan). To minimize sample variation, the mRNA expression of the target gene was normalized relative to the expression of the housekeeping gene *actin*. The quantification of mRNA levels was based on the method of Livak and Schmittgen [33].

### Statistical methods

Each treatment had at least 30 flowers with 10 flowers as a replicate. Measurements for the GA_1+3_ and IAA contents, and qRT-PCR were performed using at least three biological replicates with two technical replicates. Data were statistically analyzed using analysis of variance (ANOVA), and tested for significant (*P<0.05*) treatment differences by Tukey’s test.

## Results

### CKs induce parthenocarpic fruit set and development

**Figure 1:**
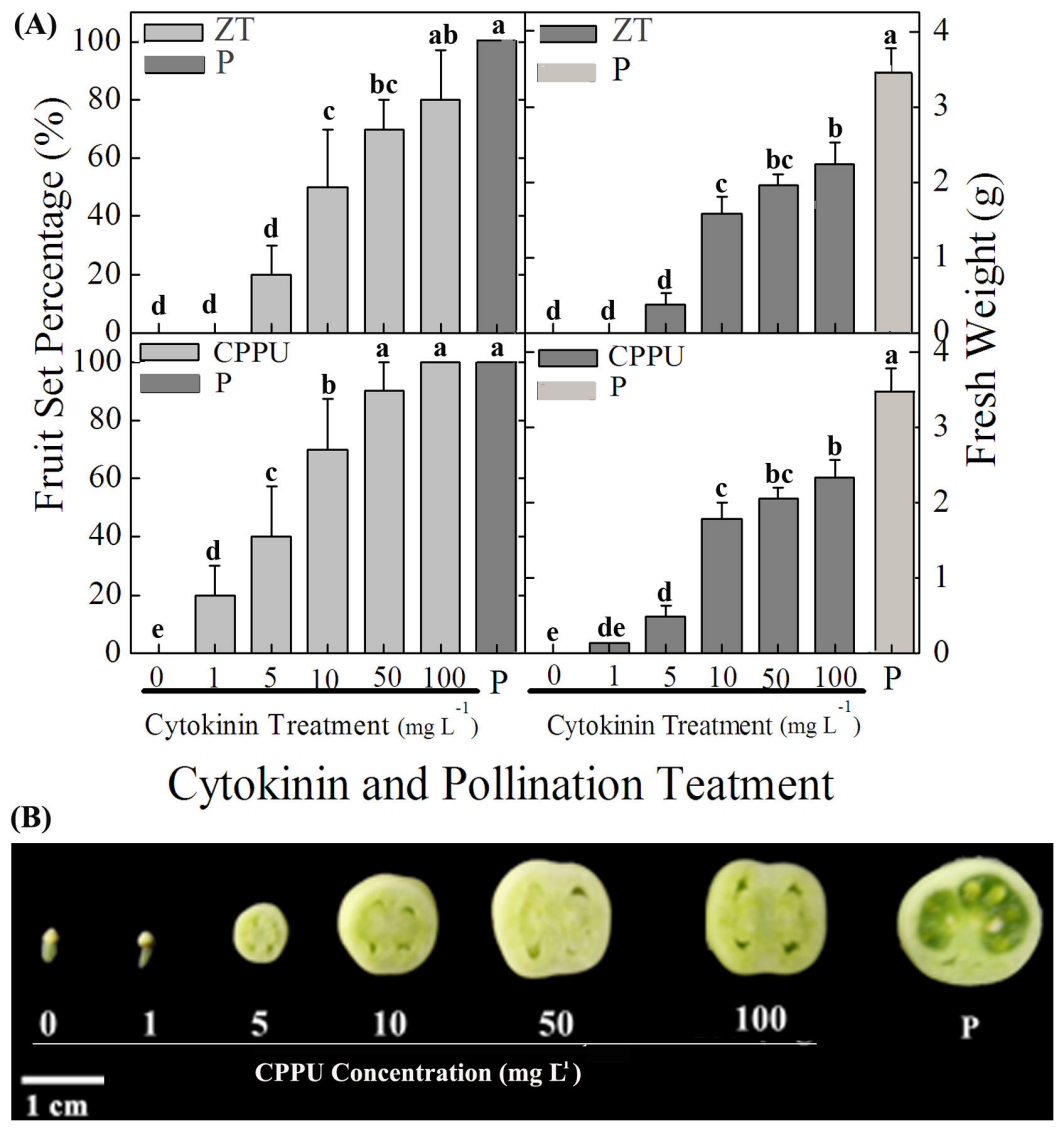
Dose response of unpollinated tomato ovaries to CPPU and ZT treatment (10 µl per ovary). (A) Fruit set percentage and fruit fresh weight (FW) in response to CPPU and ZT. (B) Transverse sections of fruits treated with CPPU. CPPU and ZT were applied at anthesis. Fruits were collected 20 days after CPPU and ZT application. Values of fruit set percentage and fruit weight are means of three replicates with 10 ovaries as a replicate (±SD). Significant differences (P<0.05) between treatments are indicated by different letters according to Tukey’s test.

To investigate whether CKs can induce parthenocarpic fruit growth in tomato, we applied *t*-zeatin (ZT) and 1-(2-chloro-4-pyridyl)-3-phenylurea (CPPU) each at 1, 5, 10, 50 and 100 mg L^-1^, which is equal to a dose of 10, 50, 100, 500 and 1000 ng per ovary, respectively, to unpollinated ovaries of tomato plants at anthesis. The unpollinated ovaries treated with water alone (served as a control) were unable to grow (Figure 1A, B), whereas fruit set percent of pollinated ovaries was near 100%. In sharp contrast with the control, both ZT and CPPU were able to induce parthenocarpic growth at all the concentrations tested, percentage of fruit set and fruit fresh weight (FW) was, however, found to be increased with a dose-dependent manner. Moreover, applications of ZT and CPPU at 100 mg L^-1^ were found to be most effective for fruit set, with a percentage of fruit set of 80% and 100%, respectively (Figure 1A). The higher efficacy of CPPU over ZT led us to choose this cytokinin for our further research.

**Figure 2:**
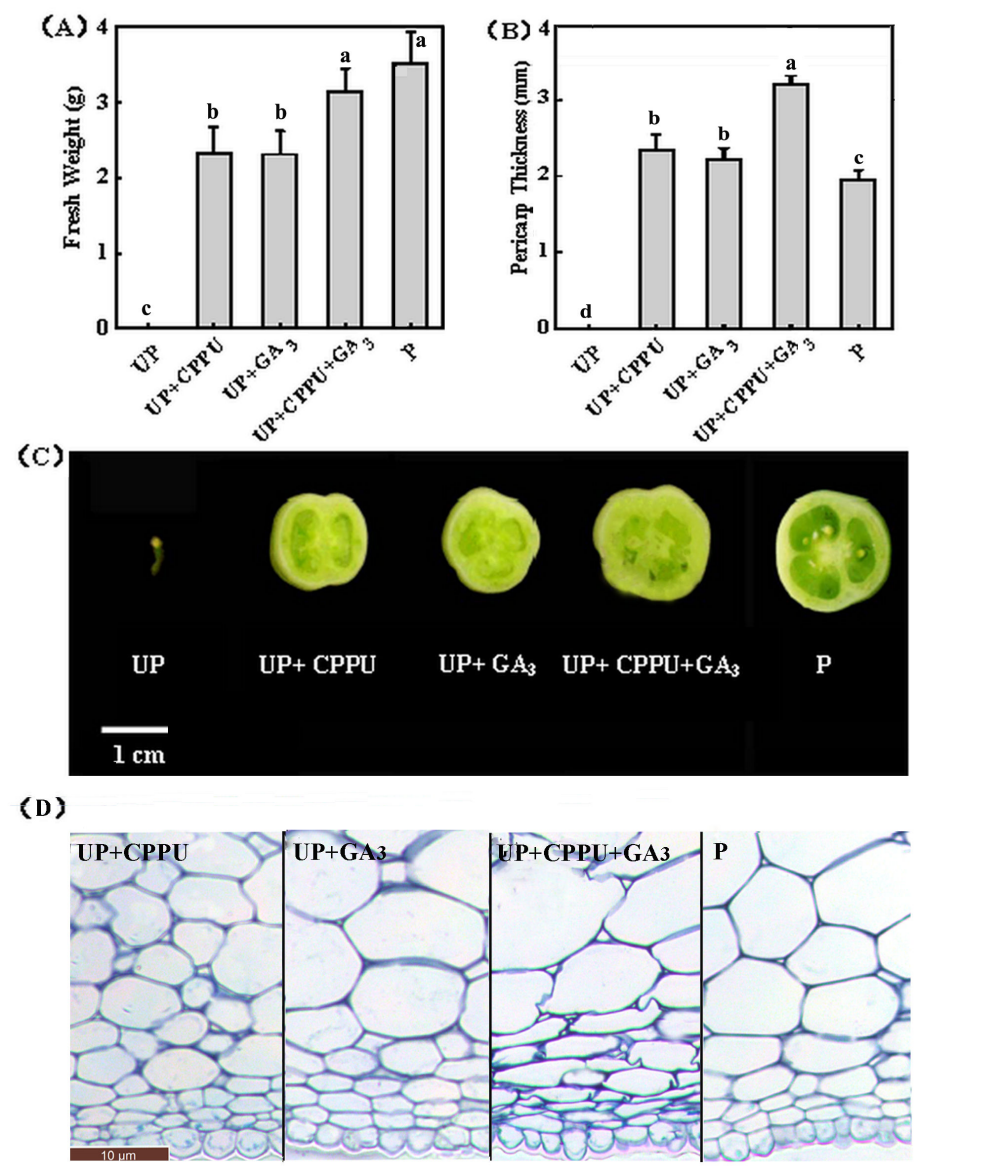
Response of unpollinated tomato ovaries to CPPU and GA_3_ treatment (10 µl/ovary; Concentration of CPPU 100 mg L^-1^ and GA_3_ 200 mg L^-1^). (A) Response of fruit FW to GA_3_, CPPU or GA_3_ plus CPPU. (B) Response of fruit pericarp thickness to GA_3_, CPPU or GA_3_ plus CPPU. (C) Transverse sections of fruit treated with GA_3_, CPPU or GA_3_ plus CPPU. CPPU and ZT were applied at anthesis. (D) Microscopic analysis of the pericarp in 20 DAA fruits. UP, unpollinated; P, pollinated. Fruits were collected 20 days after CPPU and/or GA_3_ application. Values are means of three replicates with 10 ovaries as a replicate (±SD). Significant differences (P<0.05) between treatments are indicated by different letters according to Tukey’s test.

In the following study, we compared the efficacy of CPPU (100 mg L^-1^) and GA_3_ (200 mg L^-1^) on the induction of parthenocarpic fruit. Both CPPU and GA_3_ treatments induced seedless fruits (Figure S1). The weights of both GA_3_-and CPPU-induced parthenocarpic fruits were less than that of pollination-induced fruits, whilst the pericarp thickness of CPPU-induced fruit was comparable to those of GA_3_-induced fruits (Figure 2A). Co-application of CPPU and GA_3_ showed additive effects on the fruit weight, with fruit weight increased by ca. 35% as compared with CPPU or GA_3_ alone. In addition, the pericarp was thicker than those recorded for application of CPPU and GA_3_ alone or pollination treatment due to the larger cells in the pericarp (Figure 2B, C, D).

### Parthenocarpic fruit set induced by CK contains high levels of GA_1+3_

**Figure 3:**
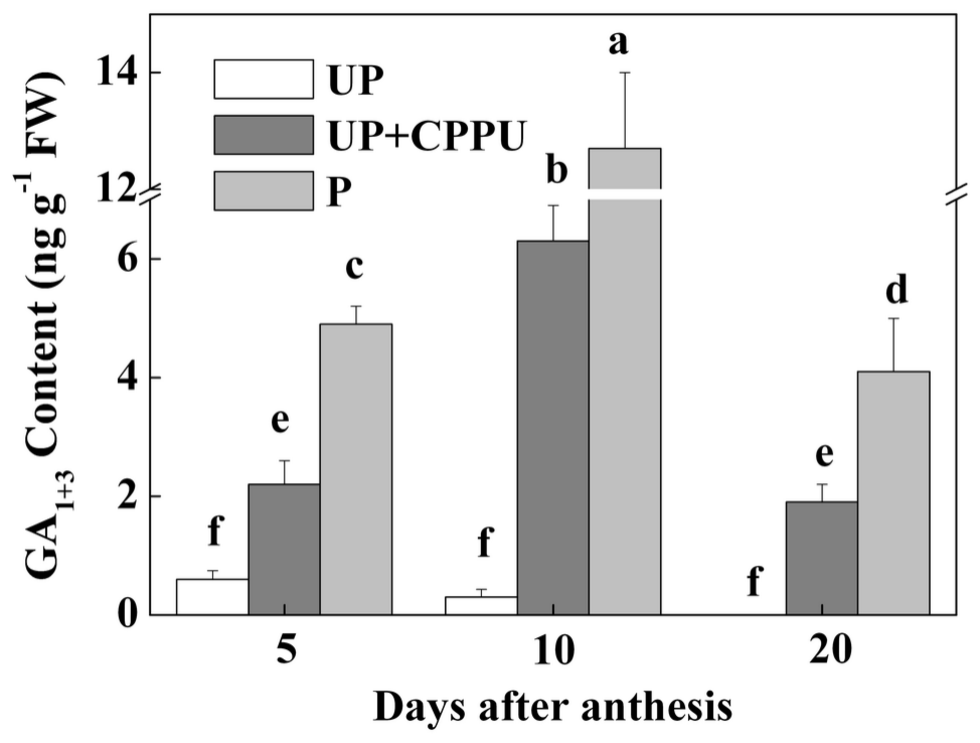
Changes in the concentration of active GA (GA_1+3_) (ng g^-1^ FW) in the CPPU-induced and pollinated fruits. All the ovaries and fruits were collected at 5, 10, 20 DAA, and applied with CPPU (10 µl/ovary, concentration of CPPU 100 mg L^-1^). UP, unpollinated; P, pollinated. All experiments were performed with three biological replicates and two technical replicates. Significant differences (P<0.05) between treatments are indicated by different letters according to Tukey’s test.

To determine whether CPPU-induced fruit set in tomato was dependent on GAs, we analyzed the dynamics of endogenous GA_1+3_ in unpollinated ovaries, pollinated ovaries and unpollinated ovaries with the application of CPPU at 100 mg L^-1^. There was no significant change in GA_1+3_ content after anthesis in the unpollinated ovaries. In contrast, GA_1+3_ contents increased sharply at 5, 10 and 20 days after anthesis (DAA) in pollinated ovaries. A significant increase in GA_1+3_ content was also recorded for CPPU-treated ovaries at 5, 10 and 20 DAA, although the increase in GA_1+3_ content in CPPU-treated ovaries was less remarkable when compared with pollinated ovaries (Figure 3). These results suggest that the increase in GAs content in CPPU treated ovaries was involved in parthenocarpic fruit set.

### GA biosynthesis inhibitor reduces CK-induced parthenocarpic fruit growth

**Figure 4:**
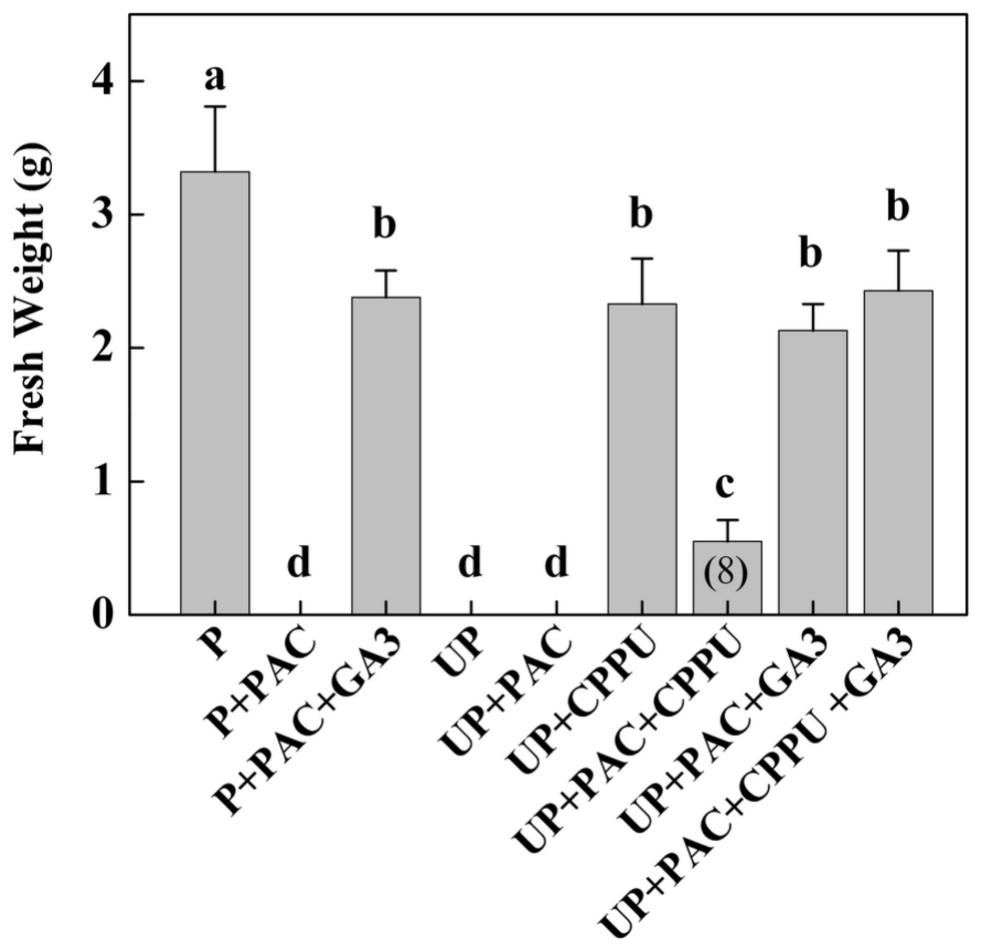
Ovary growth as influenced by pollination, CPPU, GA_3_ and PAC treatment. CPPU and GA_3_ were applied at anthesis. UP, unpollinated; P, pollinated. Ovaries were collected 20 days after hormone application. Values are means of three replicates with 10 ovaries as a replicate (±SD). Significant differences (P<0.05) between treatments are indicated by different letters according to Tukey’s test. Figures in brackets indicate the numbers of fruits developed when less than ten.

To determine the role of GAs in CPPU-induced fruit set and fruit development, we applied paclobutrazol (PAC), a GA biosynthesis inhibitor to pollinated and unpollinated ovaries with or without GA_3_ and CPPU through soil drench. The PAC application alone completely inhibited growth of pollinated ovaries (Figure 4). The PAC-induced abortion in pollinated ovaries was, however, restored by the co-application of GA_3_. In contrast, CPPU was found to be much less effective in inducing parthenocarpic fruit growth than GA_3_ when the plants were co-applied with PAC. Furthermore, the co-application of PAC, GA_3_ and CPPU induced pathenocarpic fruit with the fresh weight comparable to that induced by CPPU. These results showed that GA biosynthesis plays an important role in CPPU-induced parthenocarpic fruit set and growth in tomato.

### CK increases the endogenous level of GA with up-regulation of GA biosynthesis genes

**Figure 5:**
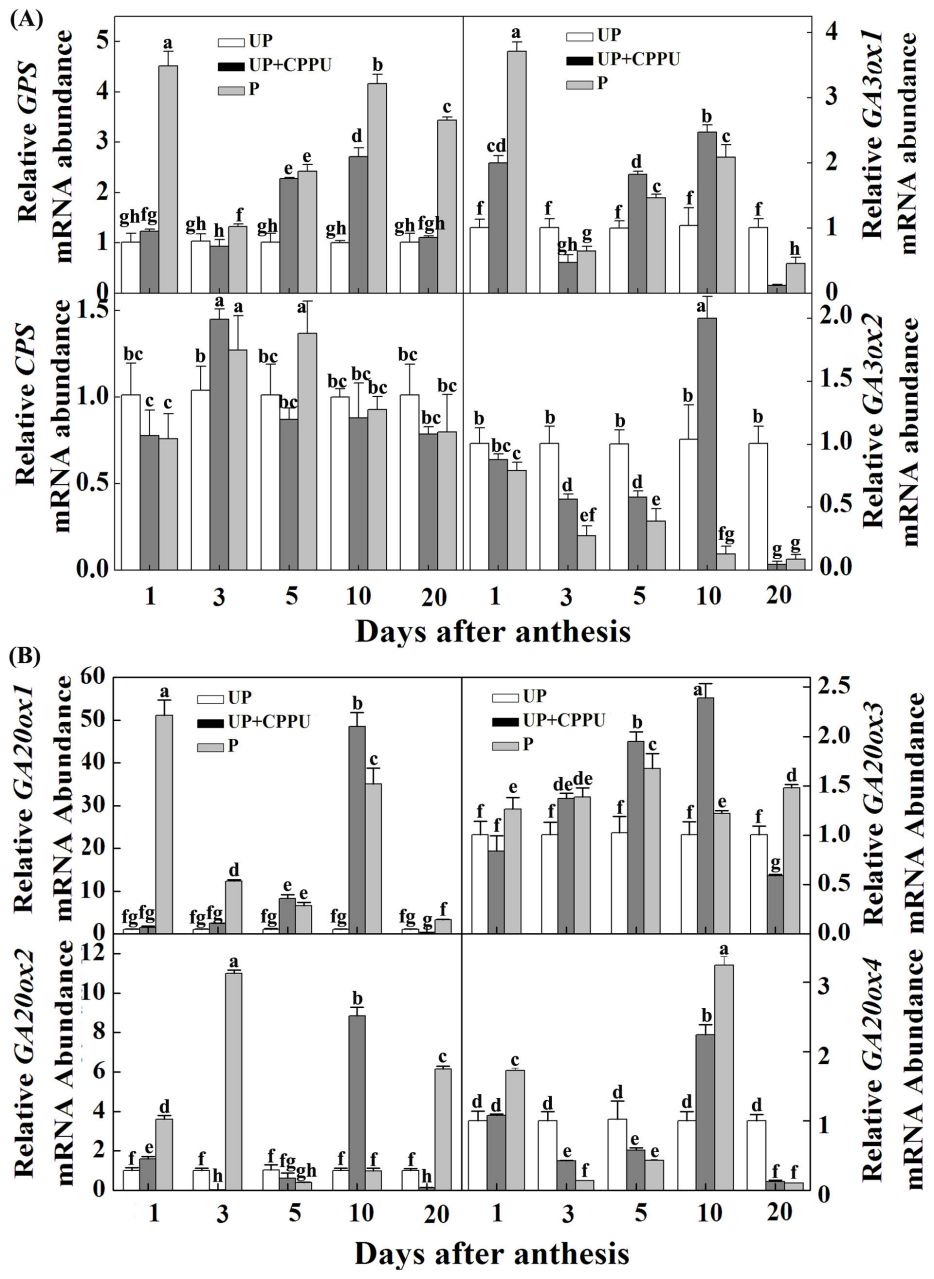
Transcript level of genes encoding enzymes of GA biosynthesis pathway as influenced by pollination and CPPU treatment. (A) *SlGPS*, *SlCPS*, *SlGA3ox1* and *SlGA3ox2* genes. (B) *SlGA20ox1*, *SlGA20ox2*, *SlGA20ox3* and *SlGA20ox4* genes. UP, unpollinated; P, pollinated. CPPU (10 µl/ovary, concentration of CPPU 100 mg L^-1^) was applied at the anthesis. Equivalent to anthesis was set as 0 d. All experiments were performed with three biological replicates and two technical replicates. Significant differences (P<0.05) between treatments are indicated by different letters according to Tukey’s test.

To examine whether CPPU-induced increase in GA pools of unpollinated fruit was related to alteration of expression of genes involved in GA metabolism, qRT-PCR analyses were performed at 1, 3, 5, 10, 20 DAA. We first investigated the transcripts of genes involved in GA biosynthesis. Pollination and CPPU treatments differentially influenced the transcripts of these genes. Generally, pollination and CPPU treatments up-regulated the transcripts of *SlGPS* (geranyl pyrophospahe synthase), *SlGA3ox1* [3b-hydroxylase (3OH-1)], *SlGA20ox1* (gibberellin 20-oxidase-1), *SlGA20ox2* (gibberellin 20-oxidase-2), *SlGA20ox3* (gibberellin 20-oxidase-3), and *SlGA20ox4* (gibberellin 20-oxidase-4) but affected the transcript of *SlCPS* (copalyl diphosphate synthase) to a less degree. Interestingly, such changes in transcripts were dynamic and pollination induced the transcripts of the many genes such as *SlGPS*, *SlGA3ox1* and *SlGA20ox* earlier than CPPU. In contrast, down-regulation of *SlGA3ox2* [3b-hydroxylase (3OH-2)] was observed for all time points analyzed with the exception of CPPU-treated ovary at 10 DAA (Figure 5A).

**Figure 6:**
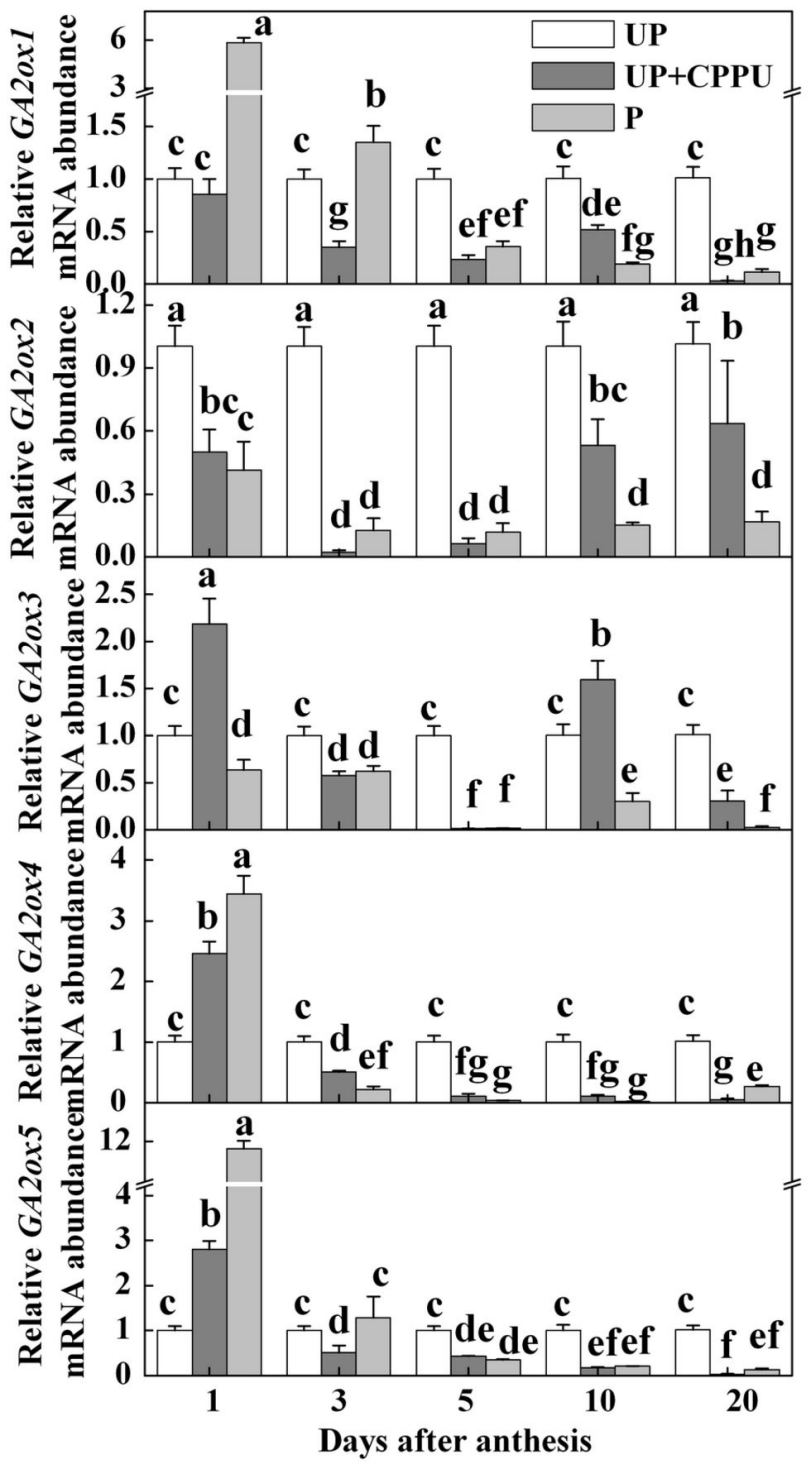
Transcript levels of genes encoding enzymes of GAs catabolism in unpollinated, pollinated and CPPU-induced parthenocarpic fruits. CPPU (10 µl/ovary, concentration of CPPU 100 mg L^-1^) was applied at the anthesis. UP, unpollinated; P, pollinated. Equivalent to anthesis was set as 0 d. All experiments were performed with three biological replicates and two technical replicates. Significant differences (P<0.05) between treatments are indicated by different letters according to Tukey’s test.

We then analyzed the transcripts of *SlGA2ox1*, *SlGA2ox2*, *SlGA2ox3*, *SlGA2ox4* and *SlGA2ox5* genes (gibberellin 2-oxidase 1-5) which encode GAs inactivating GA2-oxidases, in unpollinated, pollinated and CPPU-treated unpollinated ovaries. Increased transcripts of these genes were observed in pollinated or CPPU-treated ovaries at 1 DAA, except for *SlGA2ox2*. However, GA inactivation genes showed down-regulation in pollinated and CPPU treated ovaries at 3, 5, 10 and 20 DAA with the exception of *SlGA2ox2* and *SlGA2ox*5 at 3 DAA. The transcripts of *SlGA2ox2* showed decreases of 89% and 94% in pollinated and CPPU-treated ovaries at 5 DAA, respectively (Figure 6). These results suggest that, like pollination, CPPU could increase GAs content in the fruits at least in part through the induction of GA biosynthesis genes and suppression of GA inactivation genes.

### CK alters expression of GA-responsive and signal transduction genes

**Figure 7:**
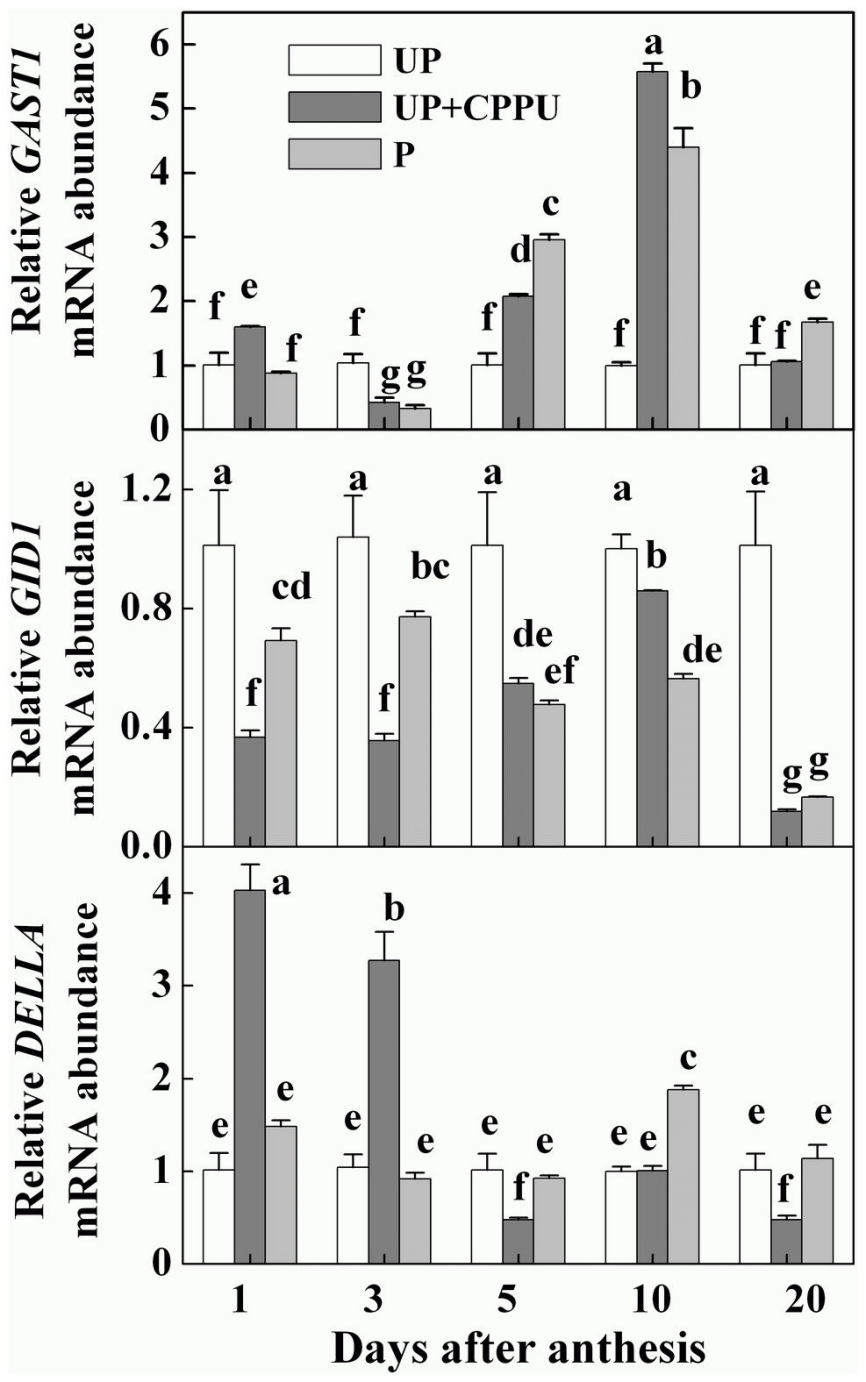
Transcript levels of genes encoding GA receptor and responsive genes in unpollinated, pollinated and CPPU-induced parthenocarpic fruits. CPPU (10 µl/ovary, concentration of CPPU 100 mg L^-1^) was applied at the anthesis. UP, unpollinated; P, pollinated. Equivalent to anthesis was set as 0 d. All experiments were performed with three biological replicates and two technical replicates. Significant differences (P<0.05) between treatments are indicated by different letters according to Tukey’s test.

To examine the crosstalk between CKs and GAs during fruit set and subsequent fruit development in tomato, we analyzed the effects of pollination and CPPU treatments on the expression of several putative GAs response and signal transduction genes in tomato. The transcript of *Gibberellin-stimulated transcripts1* (*SlGAST1*) was induced in pollinated fruits and CPPU-induced fruits. About 2-fold and 6-fold increase in *SlGAST1* transcript at 5 and 10 DAA in CPPU-induced fruits over untreated control were recorded. However, the expression of *SlGID1* (a homologue of GA receptor *GA-INSENSITIVE DWARF1*) was found to be inhibited at all stages of pollinated fruits and CPPU-induced parthenocarpic fruits (Figure 7). The expression level of *SlDELLA*, a negative regulator of GA signaling, was increased by CPPU applications at 1 and 3 DAA over the untreated control. However, *SlDELLA* transcript after 3 DAA was much lower than the untreated control. In contrast, no significant change in the *SlDELLA* transcript was observed in the pollinated ovaries (Figure 7) with the exception at 2 DAA.

### Parthenocarpic fruits induced by CK contain high level of IAA

**Figure 8:**
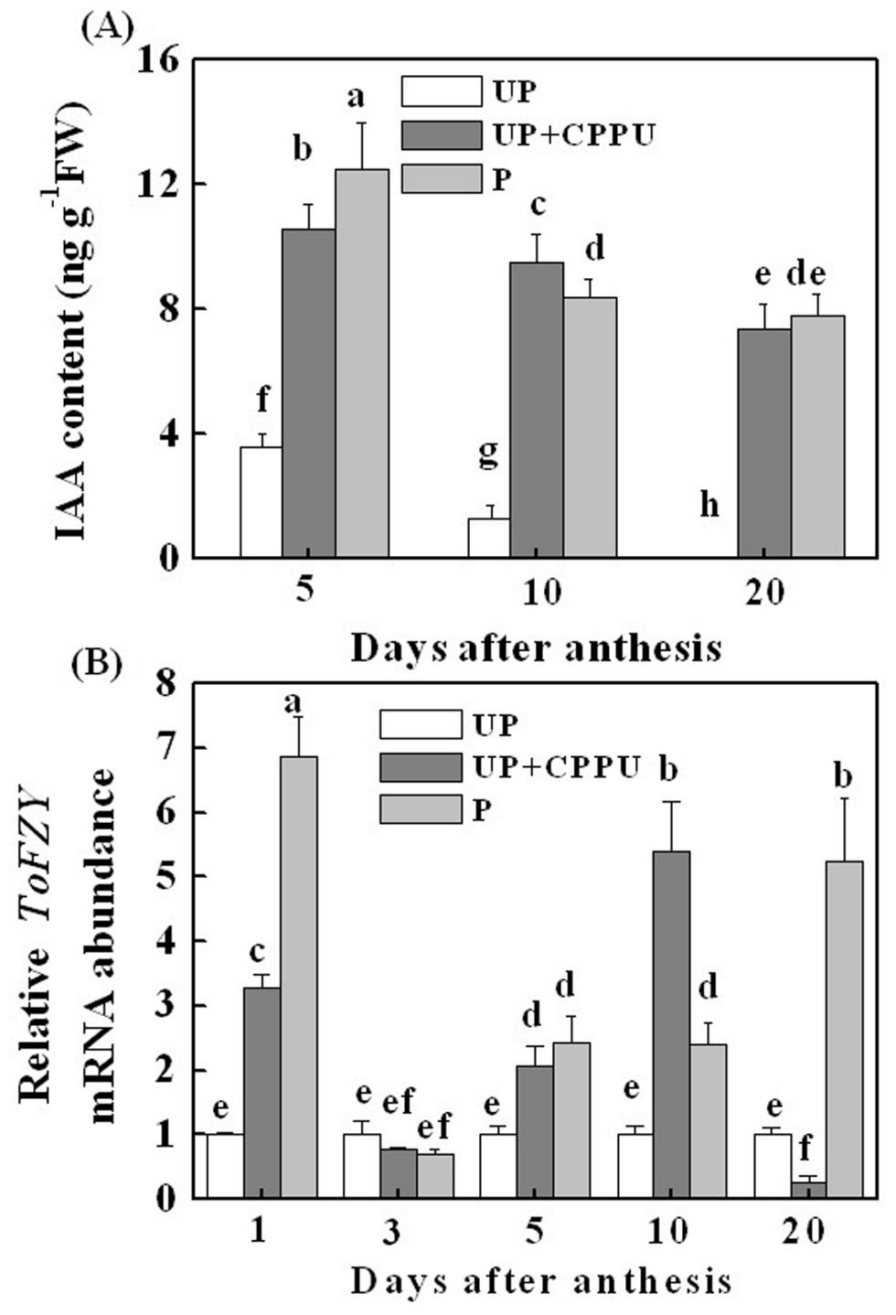
Changes in IAA concentration (A) and transcript levels of *ToFZY* (B) as influenced by pollination and CPPU treatment in the ovaries. CPPU (10 µl/ovary, concentration of CPPU 100 mg L^-1^) was applied at the anthesis. UP, unpollinated, P, pollinated. Equivalent to anthesis was set as 0 d. All experiments were performed with three biological replicates and two technical replicates. Significant differences (P<0.05) between treatments are indicated by different letters according to Tukey’s test.

To determine whether Aux biosynthesis could play a role in the CPPU induction of parthenocarpic fruit, the endogenous IAA concentration was determined. As shown in Figure 8A, the concentration of IAA in the pollinated and CPPU-treated ovaries was 3-fold and 8-fold higher than the untreated control at 5- and 10 DAA, respectively. IAA was undetectable in unpollinated ovaries at 20 DAA. In addition, the concentration of IAA in CPPU-induced fruits was decreased after 10 DAA (Figure 8A).

To examine whether the IAA accumulation in pollinated and CPPU-treated unpollinated ovaries was due to the alteration of IAA biosynthesis, we investigated the expression level of *ToFZY*, a gene encoding flavin monooxygenase, which is involved in the tryptophan dependent Aux biosynthesis pathway. The *ToFZY* expression in CPPU-induced fruits was induced intensely at 1, 5 and 10 DAA by 2-10 folds in comparison with control during the experiment (Figure 8B). These results suggest that CPPU application to unpollinated ovaries could increase the content of endogenous IAA by enhancing the transcription activity of *ToFZY*.

## Discussion

The Aux and GAs have been well documented for their capacity in inducing parthenocarpic fruit set in tomato plants [1,7,13]. Our studies showed that CKs may induce parthenocarpy in tomato partially through modulation of GA and IAA metabolisms. This conclusion is based on the following evidences: (1) CPPU, an active CK, could induce fruit set efficiently when applied directly to the unpollinated ovaries; (2) the effect of CPPU on parthenocarpy was compromised when co-applied GA biosynthesis inhibitor (PAC); (3) CPPU could enhance the biosynthesis of GA and Aux; (4) The similarity of the transcripts induced by CPPU, pollination and Aux induction.

It has been revealed that there was a significant increase in the content of endogenous hormones such as Aux, GAs and CKs in tomato ovaries after pollination [3]. In general, genotypes with parthenocarpic capacities usually own higher Aux or GAs contents in the ovary at anthesis [7,8]. In agreement with these observations, exogenous applications of Aux and GAs have been proved to induce parthenocarpic fruit set and growth in tomato [3]. Although an increase in CKs content occurs earlier than that in GAs in pollinated ovaries [3], it remains unclear that whether CKs could induce parthenocarpy or increase GAs in tomato, a model plant species for fruit development research. The data presented in our study elucidated that parthenocarpic fruit could be induced by both ZT and CPPU with the effect comparable to that of GA_3_, suggesting a role of CK in the fruit development in tomato.

In this study, we found that fruit set and development in tomato were largely dependent on the endogenous accumulation of GAs (Figure 3). There was a significant increase in GAs level in the ovaries after pollination. The application of PAC on pollinated ovaries resulted in poor ovary growth and decreased GAs accumulation, indicating that tomato fruit set and development depend on GAs synthesis after pollination and fertilization [7,12]. There is also evidence that parthenocarpy in tomato induced by Aux application was mediated through GAs [17]. In agreement with this, we found that the development of the pollinated ovary was inhibited by the application of PAC and the effect could be rescued by the co-application of GA_3_ (Figure 4). Moreover, CPPU application on unpollinated ovaries showed increased GA_1+3_ accumulations (Figure 3). Our studies showed that GA_3_ but not CPPU could completely reverse the effects of PAC in pollinated ovaries. Here, it appears that induction of parthenocarpy by CKs in tomato is partly dependent on the accumulation of GAs.

Until now, a subset of GAs has been identified from different plants including tomato fruits. A recent study showed that both GA_1_ and GA_3_ are two active forms of GAs and play an important role in fruit development in tomato [3]. Here, we showed significantly higher content of GA_1+3_ in pollinated and CPPU-treated ovaries over the unpollinated ovaries. These results suggest that CPPU, like Aux, may also participate in regulation of expression of GA metabolism genes and concomitant endogenous GA contents. The gene expression analyses showed that the transcription of genes encoding GPS, GA20ox1, GA20ox2, GA20ox3 and GA3ox1 enzymes of the GA biosynthetic pathway was largely up-regulated by pollination and CPPU treatment over the untreated control (Figure 5). Similar results were also observed in pollinated and Aux-induced fruits [17]. The expression of *GA2ox* (a multigene family) is associated with the regulation of active GA pool in plants [34] and the expression alterations of *GA2ox* genes could result in either reduced or enhanced concentration of active GAs in plants [8]. In the present study, we observed down-regulation of *GA2ox1, GA2ox2, GA2ox3, GA2ox4* and *GA2ox5* in pollinated and CPPU-treated ovaries at 3-, 5-, 10- and 20 DAA, suggesting that the downregulation in the expression of GA inactivation genes might be responsible for the coordinated increase in the concentration of active GA pool in CPPU-induced and pollinated fruits (Figure 6). All these results led us to propose that CKs enhance the GA pool via activation of GA biosynthesis genes and suppression of GA inactivation genes.

GAs and CKs have been shown to exhibit antagonistic effects on numerous developmental processes [35–37], however, the relationships between CKs and GAs in fruit development remain unknown. In this study, we found that transcript of *SlGAST1* (*Gibberellin-stimulated transcripts1*) was induced by pollination and CPPU whilst transcripts of *SlGID1*, a gene encoding a putative GA receptor [38] were downregulated in pollinated and CPPU-treated ovaries. The CPPU treatment and pollination was found to increase GAs level in the ovaries as observed for *AtGID1* in *Arabidopsis* seedlings [39]. The upregulation of *GAST1* was in agreement with those observed in GA_3_ and 2,4-D-treated ovaries [17]. In contrast, up-regulation of *SlDELLA* was observed in our study and this was especially apparent at the first days after the CPPU treatment due to the antagonistic effect of CK on the GAs signaling (Figure 7 [35–37]).

There are intensive crosstalks between phytohormones in plant growth, development and stress responses. Parthenocarpy in tomato induced by Aux application was mediated through GAs [17]. Following the up-regulation of transcripts of Aux biosynthesis genes (*ToFYZ*), we observed increased IAA level in pollination and CPPU-induced fruits over untreated control (Figure 8). These results are in agreement with those of Ohara et al. [27], who found that CPPU-treated kiwi fruit exhibited increased level of endogenous IAA. The Aux signal transduction depends on the degradation of the transcriptional regulators Aux/IAAs, although the function of most of them is not clear yet. The expression level of Aux/IAA genes (*IAA1*, *IAA2*, *IAA8*, *IAA9* and *IAA14*) was increased in response to the 2,4-dichlorophenoxyacetic acid treatment in tomato ovaries [17]. However, loss of function of *SlIAA9* exhibited parthenocarpic capability [40], suggesting that the *IAA9* transcript level may not be correlated with the protein level or, alternatively, that Aux mediated parthenocarpic induction is not mediated by *IAA9* downregulation [17]. Furthermore, it is unclear whether the expression of GA metabolism genes and associated GAs accumulation are altered by these Aux signaling components, and if so, what is the mechanism. Until now Aux and CKs mutants or chemical inhibitor for their biosynthesis are not available in tomato [17], we failed to elucidate the relation of CKs and Aux with similar methods used to study the relation of the CKs and GAs. The similarity of the transcript profile in CPPU-induced fruit (Figures 5, 6, & 7) and that observed in Aux-induced fruit [17] also suggests that CKs may induce fruit set in a similar way or in an Aux-dependent manner. It is plausible that CKs-induced parthenocarpic growth was attributable to the interaction of CKs, Aux and GAs. CKs could alter their endogenous profiles by modulating expression of the GAs/Aux metabolism genes, and then induce parthenocarpic fruit set in tomato.

It is worth noting that many genes showed different expression timing and pollination could induce the transcripts of many GAs and IAA biosynthesis and catabolic genes earlier than CPPU treatment (Figures 5-8). Generally, the locular gel in the ovaries had higher transcripts of GA and IAA biosynthesis and catabolic genes than the pericarp [17]. It is high likely that fertilization, which occurs within the locular gel triggered the transcripts of these genes to a greater degree as compared to CPPU treatment which was applied onto the pericarp. The thicker pericarp in UP + CPPU + GA_3_ treatment could also been explained by the spatial accumulation of these regulator in the fruits. Foliar application of CPPU and GA_3_ may accumulate higher in the pericarp, leading to an active cell division and cell enlargement, and finally a higher thickness of the pericarp as compared to the pollinated fruits (Figure 2D).

## Supporting Information

Figure S1 Fruit development as influenced by pollination, CPPU and GA_**3**_ treatments. Photo was taken at 40 DAA. P, pollinated.
(TIF)

Table S1 Specificity of primers used to determine the expression of IAA and GAs related genes. (TIF)
