# Supplementary figures and images for: Cytokinin-Induced Parthenocarpic Fruit Development in Tomato Is Partly Dependent on Enhanced Gibberellin and Auxin Biosynthesis
Source: PLoS One. 2013 Jul 29;8(7):e70080. doi: 10.1371/journal.pone.0070080 (PMC3726760; doi:10.1371/journal.pone.0070080)

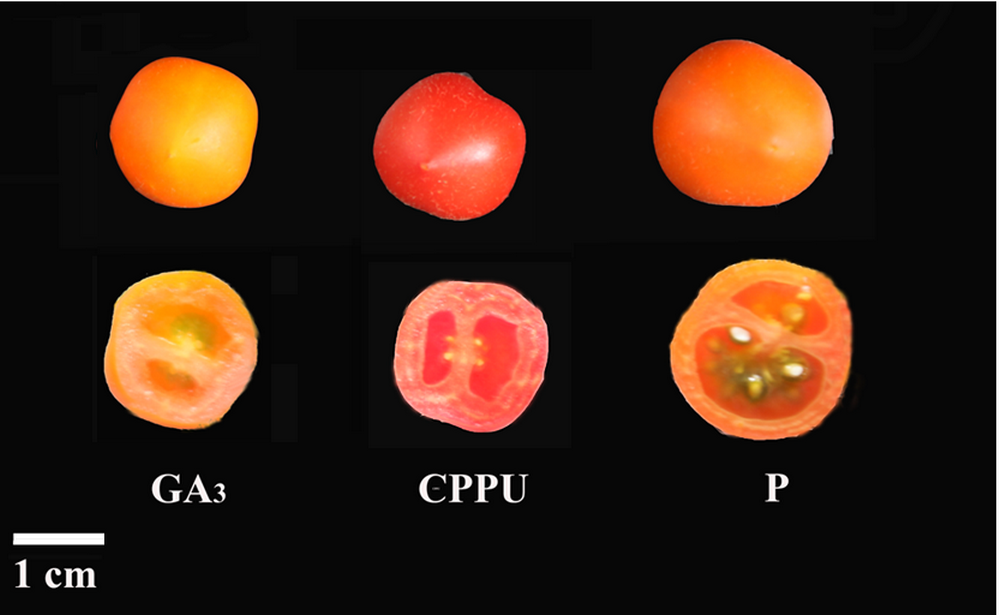

Supplement: Figure S1 — Photo was taken at 40 DAA. P, pollinated. (TIF) [file pone.0070080.s001.tif]

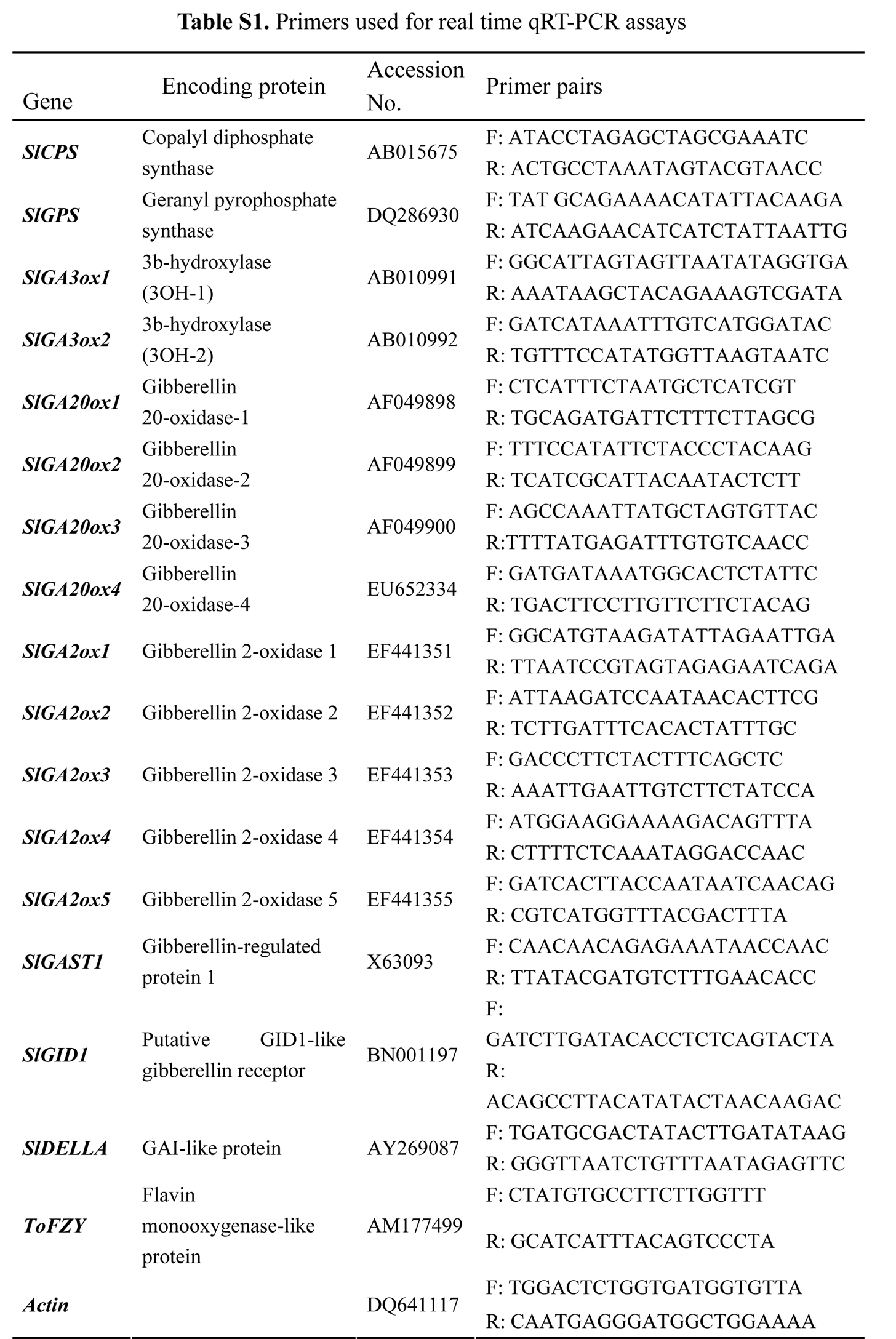

Supplement: Table S1 — (TIF) [file pone.0070080.s002.tif]
